# Genetic Evidence Supporting the Association of Protease and Protease Inhibitor Genes with Inflammatory Bowel Disease: A Systematic Review

**DOI:** 10.1371/journal.pone.0024106

**Published:** 2011-09-08

**Authors:** Isabelle Cleynen, Peter Jüni, Geertruida E. Bekkering, Eveline Nüesch, Camila T. Mendes, Stefanie Schmied, Stefan Wyder, Eliane Kellen, Peter M. Villiger, Paul Rutgeerts, Séverine Vermeire, Daniel Lottaz

**Affiliations:** 1 Department of Gastroenterology, Catholic University Leuven, Leuven, Belgium; 2 Clinical Trials Unit Bern, Bern University Hospital, Bern, Switzerland; 3 Institute of Social and Preventive Medicine, University of Bern, Bern, Switzerland; 4 Belgian Centre for Evidence Based Medicine, Katholieke Universiteit Leuven, Leuven, Belgium; 5 Department of Rheumatology, Clinical Immunology and Allergology, University Hospital of Bern, Bern, Switzerland; 6 Leuven Centre for Cancer Prevention, University Hospital Leuven, Leuven, Belgium; Universite de Montreal, Canada

## Abstract

As part of the European research consortium IBDase, we addressed the role of proteases and protease inhibitors (P/PIs) in inflammatory bowel disease (IBD), characterized by chronic mucosal inflammation of the gastrointestinal tract, which affects 2.2 million people in Europe and 1.4 million people in North America. We systematically reviewed all published genetic studies on populations of European ancestry (67 studies on Crohn's disease [CD] and 37 studies on ulcerative colitis [UC]) to identify critical genomic regions associated with IBD. We developed a computer algorithm to map the 807 P/PI genes with exact genomic locations listed in the *MEROPS* database of peptidases onto these critical regions and to rank P/PI genes according to the accumulated evidence for their association with CD and UC. 82 P/PI genes (75 coding for proteases and 7 coding for protease inhibitors) were retained for CD based on the accumulated evidence. The cylindromatosis/turban tumor syndrome gene (*CYLD*) on chromosome 16 ranked highest, followed by acylaminoacyl-peptidase (*APEH*), dystroglycan (*DAG1*), macrophage-stimulating protein (*MST1*) and ubiquitin-specific peptidase 4 (*USP4*), all located on chromosome 3. For UC, 18 P/PI genes were retained (14 proteases and 4protease inhibitors), with a considerably lower amount of accumulated evidence. The ranking of P/PI genes as established in this systematic review is currently used to guide validation studies of candidate P/PI genes, and their functional characterization in interdisciplinary mechanistic studies in vitro and in vivo as part of IBDase. The approach used here overcomes some of the problems encountered when subjectively selecting genes for further evaluation and could be applied to any complex disease and gene family.

## Introduction

About 2.2 million people in Europe and 1.4 million people in North America suffer from inflammatory bowel disease (IBD), characterized by chronic mucosal inflammation of the gastrointestinal tract. It is a lifelong disease affecting mostly young to middle aged people of 15–40 years, in a chronic and often severe way. The prevalence has increased steadily since the 1950s and is currently estimated at 0.2 to 0.3% [1], [2]. Two main phenotypes are distinguished, Crohn's disease (CD) and ulcerative colitis (UC), both with distinct histopathological features and clinical manifestations [3]. The cause of IBD is multifactorial - environmental and genetic - and poorly understood [4].

The genetic background of CD has been extensively evaluated. Since the late 1990s, a heterogeneous body of evidence on the genetics of CD has been collected by many research groups using different study designs in different settings and countries across the world. This led to significant insights into the mechanism of the disease, such as a disturbed surveillance of bacteria of the microflora by the intestinal mucosa (*CARD15*) [5], [6], dysregulation of adaptive immunity (*IL23R*) [7], or deficient autophagy (*ATG16L1*, *IRGM*) [8], [9].

The selection of genes of interest in a susceptibility region is based on subjective interpretation of external evidence, or on theoretical considerations of potential mechanisms of disease. To overcome subjective selection of candidate genes, genomic locations of genes of interest could be systematically mapped onto susceptibility regions found to be linked to or associated with IBD (“critical regions”). Genes could then be ranked according to the accumulating evidence on their association with IBD in different study types while avoiding subjective judgment.

Proteases and protease inhibitors (P/PIs) are involved in mechanisms contributing to the mucosal barrier function of the gut and may therefore be important in IBD. The Inflammatory Bowel Disease protease (IBDase) project is a collaborative project of nine academic groups across Europe funded by the European Framework Programme 7, which aims at identifying novel therapeutic targets among P/PIs. During the first stage of IBDase described here, we systematically reviewed all published genetic linkage and association studies in populations of European ancestry to identify critical genomic regions associated with IBD. We proceeded as described above to systematically map all known P/PI genes listed in *MEROPS*, a comprehensive database of peptidases [10], onto these critical regions using a computer algorithm and ranked P/PI genes according to accumulated evidence for association of P/PI genes with IBD.

## Results

**Figure 1:**
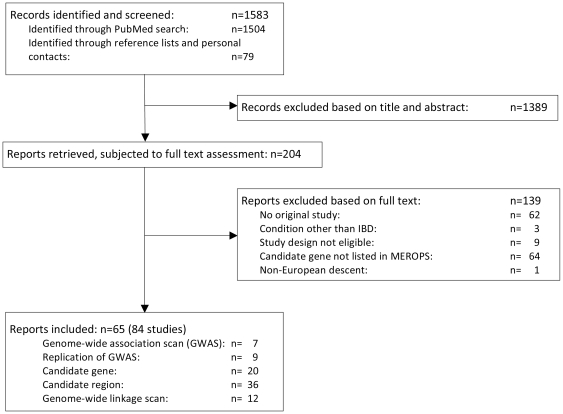
Flow diagram of the systematic review.

Figure 1 presents the flow of information through the different phases of the systematic review of genetic studies on inflammatory bowel disease in populations of European ancestry. The PubMed search resulted in 1504 hits, screening of reference lists of included papers and relevant reviews yielded an extra 79 records. We excluded 1389 articles based on information provided in title and abstract, retrieved the full texts of 204 reports, and eventually included 61 published reports and 4 unpublished reports, which were published after completion of the literature search as full journal articles [11]–[14]. These reports described 84 unique studies in the systematic review: 7 genome-wide association scans (GWAS) [8], [11], [12], [15]–[18], 9 replications of GWAS [9], [11]–[14], [16], [19], 20 candidate gene studies [8], [20]–[31], 36 candidate region studies [11], [17], [18], [32]–[66], and 12 genome-wide linkage scans [38], [42], [44], [49], [54], [59], [61], [67]–[72]. 67 studies were on CD, 37 on UC. 5 GWAS, 4 replications of GWAS, 16 candidate gene studies, 31 candidate region studies, and 11 genome-wide linkage scans studied patients with CD; 2 GWAS, 6 replications of GWAS, 8 candidate gene studies, 16 candidate region studies, and 5 genome-wide linkage scans studied patients with UC. Critical genomic regions associated with IBD were defined on the basis of the information provided in these studies, considering the HapMap of the CEU population (for further details see www.hapmap.org and methods). 38 studies that reported on patients with inflammatory bowel disease without distinction of CD and UC, and 11 studies on “mixed” families (with members affected with UC or CD), were disregarded. Table S1 presents the design and the methodological quality of included studies. 70 studies were classified to have adequate protection against bias in phenotype definition (83%), 52 against bias in genotyping (62%) and 66 against the effects of population stratification (79%).

**Figure 2:**
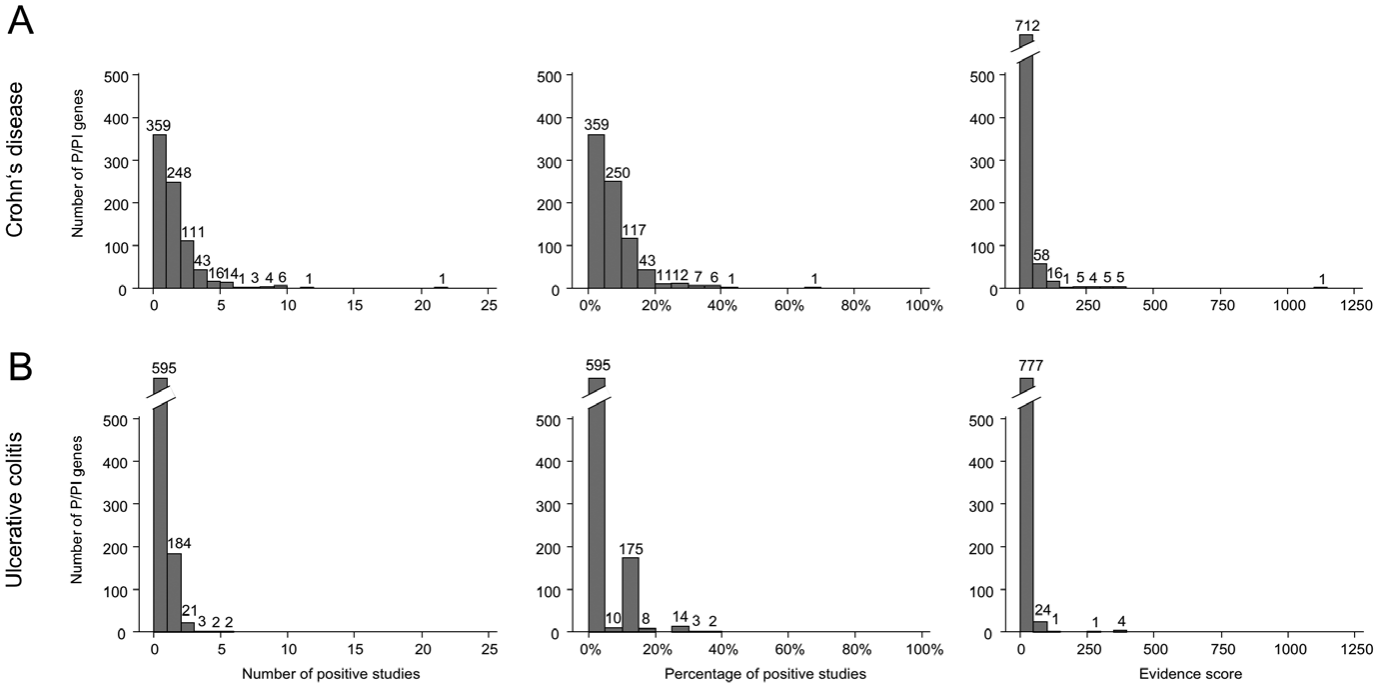
Histograms on the number of positive studies per P/PI gene (left), the percentage of positive studies per P/PI gene (middle), and the distribution of evidence scores (right) for Crohn's disease (A) and ulcerative colitis (B).

807 out of 1111 entries on P/PI genes in *MEROPS* had information on exact genomic locations available and were included (Table S2). Figure 2 presents the number of positive studies per P/PI gene (left), the percentage of positive studies per P/PI gene (middle), and the distribution of evidence scores (right) for both, CD (top) and UC (bottom). The maximum evidence score, the pre-specified primary outcome, was 1142 for CD and 363 for UC. In CD, 770 P/PI genes had evidence scores of less than 50; for 607 genes, less than 2 studies were positive. In UC, the corresponding numbers were 801 and 779. The p-value for the observed versus expected distribution of scores for associations of P/PIs with Crohn's disease was at 2.32^−70^, whereas the corresponding p-value for UC was 1.47^−42^.

### Top ranked P/PI genes in Crohn's disease

**Figure 3:**
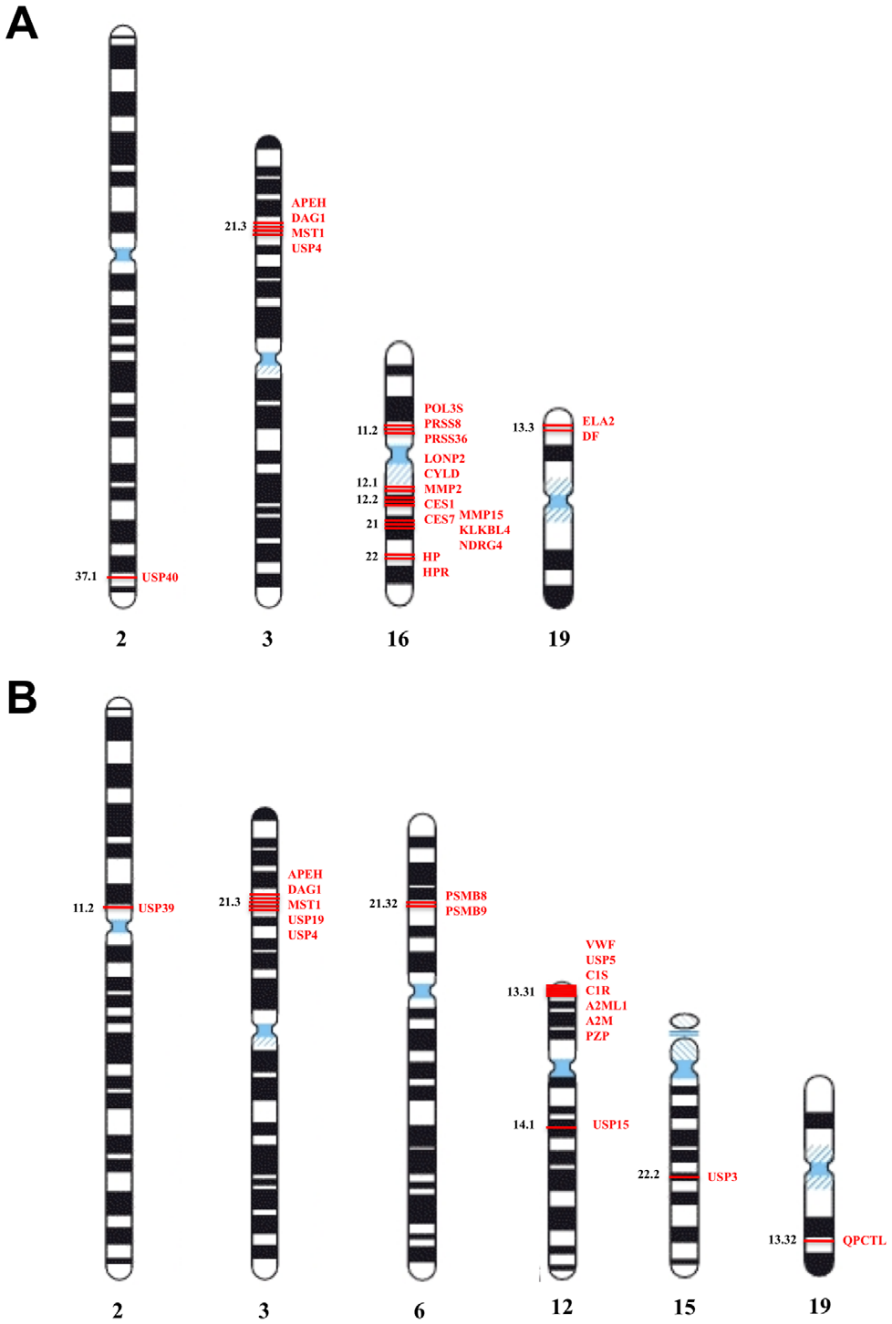
Chromosomal location of top-ranked P/PI genes in Crohn's disease (A) and ulcerative colitis (B). (A) The top 20 P/PI genes for Crohn's disease clustered on chromosomes 2 (1/20; 5%), 3 (4/20; 20%), 16 (13/20; 65%), and 19 (2/20; 10%). (B) The top 18 P/PI genes for ulcerative colitis were located on chromosomes 2 (1/18; 6%), 3 (5/18; 28%), 6 (2/18; 11%), 12 (8/18; 44%), 15 (1/18; 6%), and 19 (1/18; 6%). The depicted chromosomal banding pattern is according to Ensembl (http://Mar2010.archive.ensembl.org/Homo_sapiens/Location/View?) and has been released by the International System for Human Cytogenetic Nomenclature in 2005.

**Figure 4:**
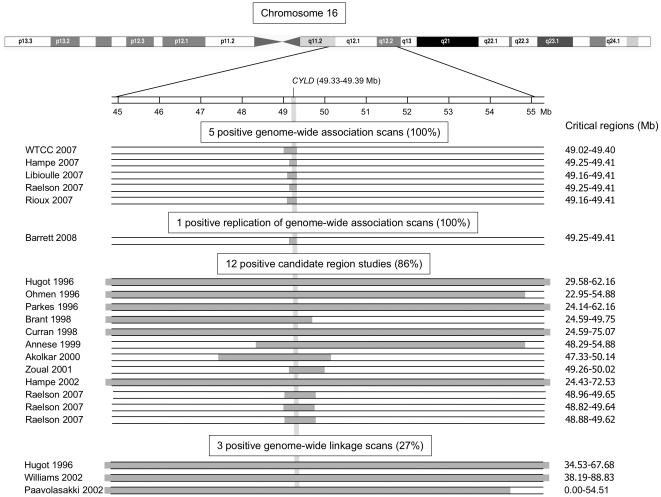
Visual display of results found in 21 positive studies of the top-ranked gene in Crohn's disease, *CYLD*. As a illustrative example for all P/PI genes, the *CYLD* (cylindromatosis/turban tumor syndrome, chr16q12.1, 49.33–49.39 Mb) gene is shown. CYLD was mapped onto the critical regions shaded in grey. The critical regions of later studies, including genome-wide association scans and replications of genome-wide association scans and some candidate region studies, were more narrow compared with critical regions of earlier studies because of the improved resolution of more recent genotyping platforms.

82 P/PI genes (75 coding for proteases and 7 coding for protease inhibitors) satisfied the threshold criteria for retention of at least 2 positive studies and evidence scores >50 and are presented in Table S3. Figure 2A presents the number of positive studies per P/PI gene (left), the percentage of positive studies per P/PI gene (middle), and the distribution of evidence scores. The largest number of positive studies was 21 (1 gene), followed by 11 (1 gene), 9 (6 genes), 8 (4 genes), 7 (3 genes), 6 (1 gene), 5 (14 genes), 4 (16 genes), 3 (43 genes), and 2 (111 genes; Figure 2A). The 20 highest ranked genes all had evidence scores >200 (Table 1). Figure 3A presents the chromosomal location of top-ranked P/PI genes in Crohn's disease: 13 out of the 20 genes were located on chromosome 16 (65%), 4 on chromosome 3 (20%), 2 on chromosome 19 (10%) and one on chromosome 2 (5%). Figure S1 provides more detailed information in a chromosome plot of the number of studies covering different genomic regions and the corresponding number of positive studies. Figure 4 presents results for the highest ranked P/PI gene, the cylindromatosis/turban tumor syndrome gene (*CYLD*) located on chromosome 16 (49.33 to 49.39 mega base pairs [Mb]), with a score of 1142 and 21 positive studies. The figure shows the width of the critical regions in 21 positive studies. *CYLD* encodes a cytoplasmic deubiquitinating enzyme interacting with cytoskeletal components and is expressed in a wide range of different tissues including the intestine. It acts as a tumor suppressor gene. Mutations, which result in a loss of function of *CYLD*, are the cause of benign tumors of skin appendages [73]–[75]. *CYLD* was followed by the acylaminoacyl-peptidase (*APEH*, chromosome 3, 49.69 Mb to 49.70 Mb), dystroglycan (*DAG1*, chromosome 3, 49.48 Mb to 49.55 Mb), macrophage-stimulating protein (*MST1*, chromosome 3, 49.69 Mb to 49.70 Mb) and ubiquitin-specific peptidase 4 (*USP4*, chromosome 3, 49.29 to 49.35 Mb) which shared the second rank with a score of 398, and 8 positive studies. The peroxisomal Lon peptidase (*LONP2*), located on chromosome 16 (46.84 to 46.94 Mb) ranked sixth with a score of 283 and 11 positive studies. From the group of matrix metalloproteases there are matrix metalloprotease-2 (*MMP2*) and membrane-type matrix protease-2 (*MMP15*), ranked 12 and 16, respectively.

**Table 1:** Top ranked P/PI genes in Crohn's disease.

Rank	Protease/protease inhibitor	Gene symbol	Genomic location^1^	Genome-wide association scans (%)^2^	Replication of genome-wide association scans (%)^2^	Candidate gene studies (%)^2^	Candidate region studies (%)^2^	Genome-wide linkage scans (%)^2^	Number of positive studies (%)	Evidence score
1	CylD protein	*CYLD*	16:49333462-49393347	5/5 (100%)	1/1 (100%)		12/14 (86%)	3/11 (27%)	21/31 (68%)	1142
2	acylaminoacyl-peptidase	*APEH*	3:49686439-49695935	1/5 (20%)	3/3 (100%)		3/6 (50%)	1/11 (9%)	8/25 (32%)	398
2	dystroglycan	*DAG1*	3:49482595-49548048	1/5 (20%)	3/3 (100%)		3/6 (50%)	1/11 (9%)	8/25 (32%)	398
2	macrophage-stimulating protein	*MST1*	3:49696393-49701110	1/5 (20%)	3/3 (100%)		3/6 (50%)	1/11 (9%)	8/25 (32%)	398
2	ubiquitin-specific peptidase 4	*USP4*	3:49290003-49352519	1/5 (20%)	3/3 (100%)		3/6 (50%)	1/11 (9%)	8/25 (32%)	398
6	peroxisomal Lon peptidase	*LONP2*	16:46835712-46944908	0/5 (0%)			8/9 (89%)	3/11 (27%)	11/25 (44%)	383
7	polyserase-3 unit 2	*PRSS53*	16:31002246-31007631	0/5 (0%)			7/8 (88%)	2/11 (18%)	9/24 (38%)	318
7	polyserase-2 unit 3	*PRSS36*	16:31057750-31068888	0/5 (0%)			7/8 (88%)	2/11 (18%)	9/24 (38%)	318
7	prostasin	*PRSS8*	16:31050255-31054320	0/5 (0%)			7/8 (88%)	2/11 (18%)	9/24 (38%)	318
10	complement factor D	*DF*	19:810665-814606	0/5 (0%)		3/3 (100%)	0/1 (0%)	2/11 (18%)	5/20 (25%)	312
10	elastase-2	*ELANE*	19:803291-807242	0/5 (0%)		3/3 (100%)	0/1 (0%)	2/11 (18%)	5/20 (25%)	312
12	carboxylesterase 1	*CES1*	16:54394267-54424576	0/5 (0%)			6/7 (86%)	3/11 (27%)	9/23 (39%)	284
12	carboxylesterase 7	*CES7*	16:54437629-54466783	0/5 (0%)			6/7 (86%)	3/11 (27%)	9/23 (39%)	284
12	matrix metallopeptidase-2	*MMP2*	16:54070589-54098101	0/5 (0%)			6/7 (86%)	3/11 (27%)	9/23 (39%)	284
15	ubiquitin-specific peptidase 40	*USP40*	2:234048905-234134623	2/5 (40%)	2/2 (100%)			0/11 (0%)	4/18 (22%)	280
16	plasma kallikrein-like protein 4	*PRSS54*	16:56871403-56886444	0/5 (0%)			5/6 (83%)	2/11 (18%)	7/22 (32%)	220
16	Membrane-type matrix metalloproteinase 2	*MMP15*	16:56616783-56638303	0/5 (0%)			5/6 (83%)	2/11 (18%)	7/22 (32%)	220
16	NDRG4 protein	*NDRG4*	16:57055118-57105024	0/5 (0%)			5/6 (83%)	2/11 (18%)	7/22 (32%)	220
19	haptoglobin-1	*HP*	16:70646009-70652458	0/5 (0%)		1/1 (100%)	2/2 (100%)	2/11 (18%)	5/19 (26%)	212
19	haptoglobin-related protein	*HPR*	16:70654624-70668645	0/5 (0%)		1/1 (100%)	2/2 (100%)	2/11 (18%)	5/19 (26%)	212

Shown are the top 20 P/PIs with an evidence score of >200 and at least 2 positive studies.

1 Chromosome: start and end boundaries (base pairs; NCBI Build 36 coordinates).

2 Number of positive studies/total studies, percentage of positive studies of respective study type in brackets; empty cells indicate no evidence available for the specific gene and the respective study type.

### Top ranked P/PI genes in ulcerative colitis

18 P/PI genes satisfied criteria for retention (14 proteases and 4 protease inhibitors, Table 2). Evidence scores for retained P/PI genes tended to be lower in UC than in CD. The highest number of positive studies was 5 (2 genes), followed by 4 (2 genes), 3 (3 genes) and 2 (11 genes; Figure 2B). None of these genes had been examined in candidate gene studies. 8 out of the 18 genes were located on chromosome 12 (44%), 5 on chromosome 3 (28%), 2 on chromosome 6 (11%) and one each on chromosomes 2, 15 and 19 (Figure 3B). Figure S1 provides more detailed information. The top 5 P/PI genes were all located on chromosome 3 within a region of 0.6 Mb: acylaminoacyl-peptidase (*APEH*, 49.69–49.70 Mb), dystroglycan (*DAG1*, 49.48–49.55 Mb), macrophage-stimulating protein (*MST1*, 49.69 0–49.70 Mb), ubiquitin-specific peptidase 4 (*USP4*, 49.29–49.35 Mb) and ubiquitin-specific peptidase 19 (*USP19*, 49.12–49.13 Mb). Four of these, *APEH*, *DAG1*, *MST1* and *USP4*, also ranked high for CD (Table 1). 8 among the 18 retained genes are linked to the ubiquitin-proteasome system (UPS): *USP4* on rank 3, *USP19* on rank 5, ubiquitin-specific peptidase 15 on rank 7 (*USP 15*, chromosome 12, 60.94–61.09 Mb), proteasome catalytic subunits 1i and 3i, and ubiquitin-specific peptidase 3, 5 and 39 on rank 8 (*PSMB9*, chromosome 6, 32.92–32.96 Mb; *PSMB8*, chromosome 6, 32.91–32.92 Mb; *USP3*, chromosome 15, 61.58–61.67 Mb; *USP5*, chromosome 12, 6.83–6.85 Mb; *USP39*, chr. 2, 85.70–85.73 Mb). Main functions of the UPS are the intracellular degradation of unneeded, damaged or toxic proteins and an involvement in antigen presentation.

**Table 2:** Top ranked P/PI genes in ulcerative colitis.

Rank	Protease/protease inhibitor	Gene symbol	Genomic location^1^	Genome-wide association scans (%)^2^	Replication of genome-wide association scans (%)^2^	Candidate gene studies (%)^2^	Candidate region studies (%)^2^	Genome-wide linkage scans (%)^2^	Number of positive studies (%)	Evidence score
1	acylaminoacyl-peptidase	*APEH*	3:49686439-49695935	1/2 (50%)	3/3 (100%)		1/4 (25%)	0/5 (0%)	5/14 (36%)	363
1	macrophage-stimulating protein	*MST1*	3:49696393-49701110	1/2 (50%)	3/3 (100%)		1/4 (25%)	0/5 (0%)	5/14 (36%)	363
3	dystroglycan	*DAG1*	3:49482595-49548048	1/2 (50%)	3/3 (100%)		0/3 (0%)	0/5 (0%)	4/13 (31%)	350
3	ubiquitin-specific peptidase 4	*USP4*	3:49290003-49352519	1/2 (50%)	3/3 (100%)		0/3 (0%)	0/5 (0%)	4/13 (31%)	350
5	ubiquitin-specific peptidase 19	*USP19*	3:49120468-49133316	1/2 (50%)	2/2 (100%)		0/3 (0%)	0/5 (0%)	3/12 (25%)	250
6	Glutaminyl cyclase-like	*QPCTL*	19:50887772-50898426	1/2 (50%)	1/2 (50%)		0/1 (0%)	1/5 (20%)	3/10 (30%)	107
7	ubiquitin-specific peptidase 15	*USP15*	12:60940454-61086165	0/2 (0%)			3/5 (60%)	0/5 (0%)	3/12 (25%)	90
8	alpha-2-macroglobulin	*A2M*	12:9096693-9160020	0/2 (0%)			1/1 (100%)	1/5 (20%)	2/8 (25%)	57
8	alpha-2-macroglobulin-like 1 protein	*A2ML1*	12:8866484-8920646	0/2 (0%)			1/1 (100%)	1/5 (20%)	2/8 (25%)	57
8	complement component activated C1r	*C1R*	12:7138294-7153069	0/2 (0%)			1/1 (100%)	1/5 (20%)	2/8 (25%)	57
8	complement component activated C1s	*C1S*	12:7038278-7048594	0/2 (0%)			1/1 (100%)	1/5 (20%)	2/8 (25%)	57
8	proteasome catalytic subunit 3i	*PSMB8*	6:32916472-32920690	1/2 (50%)	0/1 (0%)		0/3 (0%)	1/5 (20%)	2/11 (18%)	57
8	proteasome catalytic subunit 1i	*PSMB9*	6:32919891-32955342	1/2 (50%)	0/1 (0%)		0/3 (0%)	1/5 (20%)	2/11 (18%)	57
8	pregnancy-zone protein	*PZP*	12:9192704-9252233	0/2 (0%)			1/1 (100%)	1/5 (20%)	2/8 (25%)	57
8	ubiquitin-specific peptidase 3	*USP3*	15:61583863-61670712	1/2 (50%)	0/1 (0%)			1/5 (20%)	2/8 (25%)	57
8	ubiquitin-specific endopeptidase 39	*USP39*	2:85696794-85729916	0/2 (0%)			1/1 (100%)	1/5 (20%)	2/8 (25%)	57
8	ubiquitin-specific peptidase 5	*USP5*	12:6831570-6846054	0/2 (0%)			1/1 (100%)	1/5 (20%)	2/8 (25%)	57
8	von Willebrand factor inhibitor unit 2	*VWF*	12:5928301-6104097	0/2 (0%)			1/1 (100%)	1/5 (20%)	2/8 (25%)	57

Shown are the top P/PIs with an evidence score of >50 and at least 2 positive studies.

1 Chromosome: start and end boundaries (base pairs; NCBI Build 36 coordinates).

2 Number of positive studies/total studies, percentage of positive studies of respective study type in brackets; empty cells indicate no evidence available for the specific gene and the respective study type.

### Validation

In CD, all positive controls ranked among the top ranked P/PI genes. The observed evidence score for the positive control *CARD15* in CD was 1142 and 21 studies were positive. *IL23R* had a score of 430 and 7 positive studies, whereas *ATG16L1* had a score of 380 and 5 positive studies. In UC, *IL23R* had a score of 457 and 6 positive studies and would have ranked highest. The CD specific *CARD15* did not reach the pre-specified cut-off for UC, with a score of 29, and 2 positive studies. Similarly, no evidence was found for *ATG16L1* in UC. Figure S2 presents a plot of original ranks of P/PI genes against ranks yielded after omission of GWAS in a sensitivity analysis for CD (Panel A) and UC (Panel B). Results were robust for CD, but showed some changes for UC at higher ranks. All positive controls again ranked among the top ranked P/PI genes. Figure S3 presents a plot of original ranks of P/PI genes against ranks yielded after use of an alternate weighting scheme in a second sensitivity analysis for CD (Panel A) and UC (Panel B). Results were again robust for CD, but showed some changes for UC at higher ranks. Table S4 shows that 6 out of the 20 top ranked P/PI genes in CD (30%), located on chromosomes 2, 3 and 16, formally met criteria of genome-wide significance in the most recent meta-analysis of GWAS in CD [76], and Table S5 indicates that 7 out of the 18 top ranked P/PI genes in UC (39%), located on chromosomes 3 and 6, formally met criteria of genome-wide significance in the most recent meta-analysis of GWAS in UC [77]. For CD, mean evidence scores were 14 (SD 43) for negative controls and 96 (SD 180) for P/PI genes detected in at least one GWAS (difference −82, 95% confidence interval −99 to −65, p<0.001). For UC, mean evidence scores were 3 (SD 9) for negative controls and 166 (SD 143) for P/PI genes detected in at least one GWAS (difference −163, 95% confidence interval −174 to −152, p<0.001).

## Discussion

In this systematic review, computer algorithms were used to map all P/PI genes listed in the *MEROPS* database onto critical genomic regions extracted from genetic association and linkage studies performed in IBD. While the top ranked genes (Table 1 and Table 2) included some P/PIs previously found to be associated with CD and/or UC, such as *MMP2*, *MMP15* and *MST1*, a series of P/PI genes were identified, which have not been previously related to Crohn's disease or ulcerative colitis. The top 5 ranked P/PI genes for CD and UC were all characterized by high evidence scores and positive results in several GWAS and/or replication studies of GWAS. P/PI genes ranked lower were typically based on positive results in candidate region studies and genome-wide linkage scans, which were of lower resolution. At the time of the last update of our systematic review, most of the evidence had accumulated for CD, with 67 studies addressing CD as compared to 37 studies in UC. The number of positive studies among top ranked P/PIs was considerably larger, evidence scores were clearly higher and their variation more pronounced in CD as compared with UC. Unsurprisingly, ranks were completely robust for CD in a sensitivity analysis omitting GWAS, but showed some changes in the ranking for UC.

Among the top-ranked P/PIs identified in our study, some of the most promising are *CYLD* for CD, and *APEH*, *DAG1* and the group of ubiquitin-specific peptidases for both, CD and UC. In an expression microarray study, *CYLD*, encoding a deubiquitinating enzyme (also see above), has been identified as one of the most significantly downregulated genes in the intestine of IBD patients [78]. In an IBD animal model, *cyld* ^−/−^ mice displayed more severe intestinal inflammation and intestinal tumorigenesis [79]. *APEH* encodes acylpeptide hydrolase, an enzyme expressed in the intestinal mucosa, which is able to cleave N-formyl peptides derived from bacteria, a potent pro-inflammatory chemo-attractant for phagocytes [80]. *DAG1* encodes alpha- und beta-dystroglycan proteins, which are generated from a common precursor through autocatalytic cleavage. It has been hypothesized that alpha-dystroglycan acts as a receptor for *mycobacterium avium paraturbeculosis* in the intestine, a bacterium repeatedly suspected to be causally related to CD [81], [82]. The ubiquitin-proteasome system (UPS) is closely linked to the top ranked *CYLD* and includes, among the top 20 ranked genes, *USP40* for CD, *USP3*, *USP5*, *USP15*, *USP19*, *USP39*, *PSMB8*, and *PSMB9* for UC, and *USP4* for both phenotypes. It is known to play a role in the development of inflammatory and autoimmune diseases through multiple pathways, including MHC-mediated antigen presentation, cytokine and cell cycle regulation, and apoptosis [83]. Finally, *MST1*, already repeatedly associated with IBD [11], [84], [85], was also ranked high for both CD and UC. It encodes macrophage stimulating protein 1 and is involved in apoptosis. Note however that the protein is presumably not active as a protease due to a mutation at the catalytic site.

In this systematic review we included genetic studies with differences in methodology (linkage versus association) and thus differences in resolution and accuracy by which a given genomic region was studied, in genetic markers used, and in definitions applied to establish and report association or linkage of a gene or region with IBD. A formal meta-analysis was not feasible, therefore. Rather, we based our systematic review on an approach commonly referred to as vote count [86], and merely distinguished between positive and negative studies on a specific P/PI gene as identified by our mapping algorithm. The higher the power of the studies included in the systematic review the more appropriate vote count methods will be [87]. As suggested by Barrett et al. [14], individual genetic studies in IBD often have enough power to detect large effect sizes, but limited power to detect small to moderate effects corresponding to odds ratios of 1.2 to 1.5. It is therefore likely that some of the vote counts observed in included studies were false negative on small to moderate associations of a P/PI gene with IBD. We took this into account by using low cut-offs for evidence scores of P/PI genes to be retained in the final ranking. This low cut-off counteracted the limited power of individual genetic studies and was deemed to decrease the overall risk of false negative conclusions about the association of a P/PI gene with CD or UC in our review. This means that a P/PI gene was retained even if the proportion of positive studies was small. If the majority of negative studies were true negatives and the majority of positive studies false positives, we would erroneously suggest an association of a retained P/PI gene with IBD. There will always be a trade-off between false negatives and false positives, and our strategy of counteracting false negatives was bound to increase the risk of false positives. Therefore, any of the retained P/PI genes considered for further scientific investigation needs to be confirmed first in an adequately powered, independent replication study on its association with CD or UC.

We emphasize that even if associations between a P/PI gene and IBD were true, this does not necessarily indicate that a polymorphism in this gene has a causal role for CD or UC. Genetic linkages and associations are influenced by linkage disequilibrium patterns of the study population, which limit the resolution of any genetic study. Therefore, associations observed in our study may not be attributable to single genes but rather to genomic regions containing several genes, which are in strong linkage disequilibrium. Therefore, genes other than the P/PI gene identified by our algorithm in a specific critical region could be responsible for the observed association with IBD. For example, the top-ranked P/PI gene in CD, *CYLD* on chromosome 16 (49.33 to 49.39 Mb) is located adjacent to *CARD15* (Mb 49.28 to 49.32) which traces back to the same critical region. The functional link of *CARD15* to IBD has been firmly and reproducibly established [5], [88], [89]: there are several well-characterized polymorphisms in *CARD15* that lead to different capacities of the protein products to regulate NF-kappaB-mediated inflammatory responses to bacterial components in the gut, thus providing a causal explanation for the observed association with the disease. However, the association and linkage signals of the involved critical region on chromosome 16 can only partially be explained by polymorphisms in *CARD15*: Hampe et al. found that a robust association signal in this region remains after stratification by *CARD15* polymorphisms [46]. It is therefore plausible that an adjacent gene, such as *CYLD*, may account for this association signal in this critical region and the neighborhood of *CYLD* to *CARD15* should not preclude *CYLD* to be considered as a potential candidate P/PI gene and further investigated in IBD. Conditional genotypic analysis of *CYLD* in *CARD15*-negative patients, which is ongoing in the replication study, will clarify the hypothesized independent association signals in both genes.

Another important limitation is that we were unable to gauge the direction of associations between P/PI genes and IBD for two reasons. First, in the presence of identical genetic markers and definitions of associations, the vote count used in our study could not distinguish between an increase in the odds of IBD associated with the marker in one study and a decrease in the odds associated with the marker in another study. If both studies were positive on an association of this marker with IBD, then we would consider them to be concordant even though they may have found opposite directions of associations. Second, the heterogeneity in markers used in different studies makes it impossible to achieve comparability of measures of association. Even if two studies showed an association in the same direction and of a similar magnitude, differences in the types of genetic markers could still mean that the two studies are actually discordant. Ignoring the directions of associations as described here, may therefore result in an overestimation of the accumulated evidence and we emphasize once more the need for validation of our results. Although being careful in avoiding any duplicate extraction within the same genetic region of the same population, we cannot not fully exclude that some genetic region of some patients were included multiple times in our study if some previously studied patients were subsequently included in later studies of larger populations. Finally, candidate gene and candidate region studies may be subject to selective reporting and publication bias, with predominant reporting of statistically significant results. We cannot exclude that this has influenced our ranking of some P/PI genes. We believe, however, that the direction and magnitude of this bias are similar across all P/PI genes. Therefore its overall impact on relative rankings is likely to be small. In addition, a variety of strategies for internal validation through negative and positive controls suggested our approach to be valid.

Our method is complementary to the classical approach of formal meta-analysis: using the algorithm, genetic evidence can be gauged genome-widely, considering all available studies of different types, even if different analytical methods were used. The common concept ascertained is the ‘critical genomic region’ irrespective of study design and genotyping technique used. This avoids the need for fully compatible genetic markers or imputations to achieve compatibility, as used in classical meta-analysis [14], [76], [77], [90]. The ranking algorithm is based on numerical information about the critical regions and the genomic locations of P/PI genes in the human genome in relevant databases. Errors in these databases inevitably lead to errors in the gene ranking, which can only be addressed in subsequent updates. It must be noted that many entries in *MEROPS* are putative P/PI genes predicted theoretically, but have not been functionally validated. For example, Haptoglobin (*HP*) and Haptoglobin-related protein (*HRP*), which rank in the top 20 for UC (Table 2), are taken up in the *MEROPS* database due to a peptidase inhibitor sequence motif, despite that there is no supporting experimental evidence. The high scores for the firmly established susceptibility genes *CARD15*, *ATG16L1* and *IL23R* in CD, and *IL23R* in both CD and UC, which were generated by the algorithm after mapping the genomic locations of these genes onto the critical regions extracted from genetic studies, suggest that the methodology used in our systematic review is indeed valid. The scores for *CARD15*, *ATG16L1* and *IL23R* in CD, and *IL23R* in UC, were in the range of the 20 top-ranked P/PI genes in both phenotypes.

After closure of our database, various genome-wide association scans in UC and CD were published [76], [77], [91]–[94]. Several previously known genomic regions were replicated and novel susceptibility regions were revealed. These studies, together with other recently published genetic studies [95]–[100], increase considerably the available genetic information for UC and CD, and will be considered in future updates. In an attempt to validate our approach, however, we examined whether top ranked P/PI genes met genome-wide significance at the level of p<5×10^−8^ in the two most recent meta-analyses of GWAS in CD and UC [76], [77]. For both conditions, the 5 highest ranked P/PI genes all met genome-wide significance (Table S4 and Table S5). For 14 of the top 20 P/PI genes in CD and 11 of the top 18 P/PI genes in UC, criteria of genome-wide significance were not formally met in the meta-analyses [76], [77]. The relevant, but only partial concordance in 30 to 40% of P/PI genes suggests in any case that our approach is not redundant in the presence of large scale meta-analyses. Rather, it will provide complementary information to be subsequently verified. Based on published results, we are currently unable to determine whether the discordance observed was due to false negatives in the meta-analyses or false positives in our study and would welcome detailed data on all top ranked P/PI genes as found in these meta-analyses [76], [77]. As part of the EC-funded research project IBDase, the ranking of P/PI genes established in our systematic review is also used to guide replication studies of candidate P/PI genes and their functional characterization in interdisciplinary mechanistic studies in vitro and in vivo. These additional data will contribute to our understanding of putative causal links of these genes with IBD.

## Methods

### P/PI gene table

We used the *MEROPS* database, release 8.2 (August 2008) (http://merops.sanger.ac.uk) [10], which includes 694 known human protease genes and 163 protease inhibitor genes, to identify all known human P/PI genes. All entries were used, including hypothetical genes predicted by automatic algorithms. If exact megabase locations were unavailable in *MEROPS*, we obtained exact locations from the Ensembl Genome Browser [101] and the Entrez Gene database [102]. All locations referred to the National Center for Biotechnology Information (NCBI) 36 assembly of the human genome updated November 2005. In case of discrepancies, the genome draft of the Human Genome Organisation took precedence over Celera. If only chromosome numbers or information on cytobands was provided for a P/PI gene and accurate information on genomic location was lacking, the gene was dropped.

### Literature search and selection of reports

We proceeded according to a binding protocol, accessible online to members of the research consortium (www.ibdase.org). We searched PubMed to identify all relevant reports published until and including June 2008 using the search string (“Inflammatory Bowel Diseases” [Mesh] OR “Crohn Disease” [Mesh] OR “Colitis, Ulcerative” [Mesh]) AND (genome-wide association stud* [title, abstract] OR genome-wide scan* [title, abstract] OR genome scan* [title, abstract] OR genetic linkage [title, abstract] OR mutation* [title, abstract] OR polymorphism* [title, abstract] OR “genetic predisposition to disease” [MeSH]). In addition, we checked reference lists of retrieved reports, relevant narrative reviews [89], [103]–[106] and meta-analyses [14], [90]. We included genome-wide association scans (GWAS), replications of GWAS, candidate gene studies, candidate region association studies, candidate region linkage studies, and genome-wide linkage scans in patients with CD or UC, and controls of Caucasian origin. All GWAS, replication studies, candidate region studies and genome-wide linkage scans were included, irrespective of whether they had specifically reported on a P/PI gene. Candidate gene studies were included if they had studied at least one of the P/PI genes listed in *MEROPS* [10]. One report could include multiple studies, for example both a GWAS and a replication of this GWAS in a different population. These were then considered as separate studies. If multiple reports referred to the same study, we used all reports for data extraction while carefully avoiding any duplicate extraction within the same genetic region of the same population. If multiple study types were performed in the same population (for example both a GWAS and a candidate gene study), we typically considered all types since genomic locations and resolutions were different between types. Studies reported only as abstracts were excluded. Two reviewers evaluated independently reports for eligibility. Disagreements were resolved by discussion.

### Data extraction

Data were extracted by one out of three investigators (IC, GEB or EK) and checked by a second investigator. Disagreements were resolved by discussion. We extracted the measures of linkage or association with IBD as reported by the authors, the corresponding 95% confidence interval and p-values. We used the criteria specified by the authors to distinguish between statistically positive and negative results. If the authors did not specify a cut-off, we used the criteria by Lander and Kruglyak for linkage studies [107] and p<5×10^−7^ for significance in GWAS [108].

For candidate gene studies, the critical region was defined as the genomic location of the studied genes. This exact location was obtained from *MEROPS* [10], Ensembl [101] or Entrez Gene database [102] as described above. For all other study types, we referred to critical regions as defined by the authors. If information on the exact region of linkage or association was unavailable, the critical region was defined depending on the type of study. In candidate region linkage studies, we used information given on the used microsatellite markers to establish the boundaries of the critical region. These boundaries were considered to be located one score unit upstream and one unit downstream from the peak non-parametric linkage (NPL) or logarithm of odds (LOD) score. If the markers and/or NPL/LOD-scores were not provided in text or tables, we extracted the information from published graphs. For whole-genome linkage scans, the same approach was used, with the extension of defining the critical region to extend one average distance between two markers upstream and downstream if no information on NPL/LOD scores was available. For candidate region association studies using single nucleotide polymorphisms (SNPs), critical regions were defined by the position of the most upstream and most downstream significant SNP. In GWAS and replication studies of these GWAS, the critical region was determined as described by Barrett et al. [14]. In brief: The HapMap of the CEU population was used to define the set of HapMap SNPs with an r^2^>0.5 to the reported SNP. The critical region was delimited by the outer boundaries of the flanking HapMap recombination hotspots that contained this set of SNPs. If the outer SNPs in this set were residing within a recombination hotspot, the adjacent HapMap hotspot was used to define the boundary. Linkage disequilibrium (LD) data and recombination hotspot positions were retrieved from the HapMap Genome Browser, release 24 (www.hapmap.org) [109]. Coordinates for the SNP positions and recombination hotspots were in NCBI build 35 coordinates [110]. To map these regions onto the gene locations in *MEROPS*, we converted NCBI 35 coordinates to NCBI 36 coordinates using the Batch Coordinate Conversion (LiftOver) utility provided by UCSC (http://genome.ucsc.edu/cgi-bin/hgLiftOver).

The methodological quality of included studies was assessed referring to three major types of bias occurring in genetic studies [108]: bias in phenotype definition, bias in genotyping, and population stratification. Studies were classified to have adequate protection against bias in phenotype definition if clear, widely agreed definitions were used, efforts for retrospective harmonization were undertaken, or a prospective standardization of phenotypes was performed. Protection against bias in genotype definition was deemed to be adequate if appropriate quality control checks were reported. The effects of population stratification were deemed to be adequately avoided if same descent groups were included, statistical adjustment for reported descent was described, a family-based design was used, or genomic control was performed [108].

### Data synthesis

Each gene and critical region extracted from the genetic studies was specifically located on the human genome using the mega-base location of upstream and downstream boundaries as described above. For example, in a genome-wide linkage study [71], a critical region associated with IBD was described to be located at 1p32. We translated this genomic region into 51.29 mega base pairs (Mb) upstream boundary and 60.91 Mb downstream boundaries. Then, we used a computer algorithm to map all P/PI genes listed in the *MEROPS* database onto the studied critical regions: for each P/PI gene, we determined whether the location of the gene overlapped with any of the extracted critical regions evaluated in the genetic studies. In view of potential deficiencies in precision and resolution of source databases and the possibility of regulatory upstream and downstream regions located adjacent to the genes coding for the P/PI, we broadened the width of the specified P/PI gene location by 10 kilo base pairs for both the upstream and downstream boundary. For example, matrix metallopeptidase-2 (MMP-2) was defined by 54.07 Mb upstream and 54.10 Mb downstream boundary; we widened this to 54.06 Mb upstream and 54.11 Mb downstream.

For each study type, we determined the proportion of positive studies separately for CD and UC. The proportion was defined as the number of studies positive on a P/PI gene divided by the total number of studies found by the computer algorithm to assess critical regions including the P/PI gene. For *MMP2* in CD, for example, none of the 5 GWAS was positive (proportion 0.0), *MMP2* was not investigated in replications of GWAS, neither in candidate gene studies, but 6 of 7 candidate region studies were positive (proportion 0.86), and 3 of 11 genome-wide linkage studies (proportion 0.27). We pre-specified an overall “evidence score” as primary outcome of our study. The evidence score took into account both, the absolute number of positive studies, and the proportion of positive studies among the total number of available studies, as well as differences between study types in the accuracy of genetic analyses:
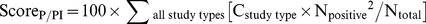
with Score_P/PI_ being the evidence score, ∑_all study types_ the sum across all study types, N_positive_ the number of positive studies on a P/PI gene, N_total_ the total number of studies found by the computer algorithm to evaluate the P/PI gene, and C_study type_ a weighting factor according to study type. Candidate gene sudies, GWAS and replication studies of GWAS were considered more accurate than candidate region and genome-wide linkage scans, therefore the weighting factor was set at C_study type_ = 1.00 for GWAS, replication of GWAS and candidate gene studies, C_study type_ = 0.50 for candidate region studies, and C_study type_ = 0.33 for genome-wide linkage scans. We ranked all P/PI genes according to this score, but discarded P/PI genes with less than 2 positive studies or a score ≤50; criteria for discarding were identical for CD and UC. An evidence score of 50 will be reached, for example, if two out of four candidate region studies were positive. Then, we derived test statistics for observed versus expected uniform distributions of scores using a signed test. As “positive controls” we used non-P/PI genes with firmly established association with CD (*CARD15* on chromosome 16q12.1 [Mb 49.28 to 49.32], *ATG16L1* on chromosome 2q37.1 [Mb 233.82 to 233.87]) and both CD and UC (*IL23R* on chromosome 1p31.3 [Mb 67.40 to 67.50]). If these positive controls ranked high this would suggest our approach to be valid. Since GWAS received major weight in the calculation of evidence scores, we performed a sensitivity analysis recalculating ranks after omission of GWAS. A second sensitivity analysis was performed using an alternate weighting scheme for different study types, with weighting factors set at C_study type_ = 1.00 for GWAS, replication of GWAS and candidate gene studies, C_study type_ = 0.75 for candidate region studies, and C_study type_ = 0.50 for genome-wide linkage scans. Then, we used repeated random sampling of P/PI genes not identified in GWAS to derive “negative controls” and compared mean scores found for these negative controls with mean scores in P/PI genes who met genome-wide significance in at least one GWAS at p<5×10^−8^. Lower mean scores in negative controls would support the validity of our approach. Finally, we determined whether top ranked P/PI genes met genome-wide significance (p<5×10^−8^) in the two most recent meta-analyses of GWAS in CD and UC [76], [77]. The data synthesis and mapping was performed using GeneRank (University of Bern, Bern, Switzerland) developed in Webspirit (2 mt software Ltd, Ulm, Germany) and Stata version 10.1 (College Station, Tex, USA).

## Supporting Information

Figure S1 **Chromosome plot of the number of studies covering different genomic regions and corresponding numbers of positive studies, presented separately for CD and UC.** The total number of performed studies is shown in grey, separately for CD (upper track) and UC (lower track), the number of positive studies reporting a genetic association with CD in blue (upper track) and the number of positive studies reporting a genetic association with UC in red (lower track). Top ranked 20 CD and UC P/PI genes are specified in the figure in blue if associated with CD, in red if associated with UC, in black if associated with both phenotypes. Critical regions defined as before were processed in 1 Mb bins with a perl script and the data was visualized using UCSC Genome Graphs (http://genome.ucsc.edu/cgi-bin/hgGenome).
(PDF)

Figure S2 **“GeneRank” Sensitivity assay.** Original ranks of P/PI genes on the x-axis are plotted against ranks yielded after omission of GWAS in sensitivity analyses on the y-axis for CD (Panel A) and UC (Panel B).
(PDF)

Figure S3 **Ranking of P/PI genes in CD and UC with different weighting factors of types of genetic studies.** Ranks obtained for CD (panel A) and UC (panel B) applying the original weighting factors set at C_study type_ = 1.00 for GWAS, replication of GWAS and candidate gene studies, C_study type_ = 0.5 for candidate region studies, and C_study type_ = 0.33 for genome-wide linkage scans (rank 1, x-axis) plotted against ranks obtained with an alternate scheme using weighting factors set at C_study type_ = 1.00 for GWAS, replication of GWAS and candidate gene studies, C_study type_ = 0.75 for candidate region studies and C_study type_ = 0.33 for genome-wide linkage scans (rank 2, y-axis).
(PDF)

Table S1 **Assessment of the methodological quality of included studies.**
(XLS)

Table S2 **Proteases and protease inhibitors with exact genomic location extracted from the Merops database (release 8.2).**
(XLS)

Table S3 **All proteases and protease inhibitors fulfilling the pre-defined thresholds for Crohn's disease (evidence score >50 and at least 2 positive studies).**
(DOC)

Table S4 **Top ranked P/PI genes in CD mapping to loci identified in the GWAS meta-analysis.** Top ranked P/PI genes in CD mapping to loci with genome-wide significance (p<5*10^−8^) identified in the GWAS meta-analysis by Franke et al. (Nature Genetics, Dec 2010).
(XLSX)

Table S5 **Top ranked P/PI genes in UC mapping to loci identified in the GWAS meta-analysis.** Top ranked P/PI genes in UC mapping to loci with genome-wide significance (p<5*10^−8^) identified in the GWAS meta-analysis by Anderson et al. (Nature Genetics, Feb 2011).
(XLSX)
