# Supplementary figures and images for: Genetic Evidence Supporting the Association of Protease and Protease Inhibitor Genes with Inflammatory Bowel Disease: A Systematic Review
Source: PLoS One. 2011 Sep 8;6(9):e24106. doi: 10.1371/journal.pone.0024106 (PMC3169567; doi:10.1371/journal.pone.0024106)

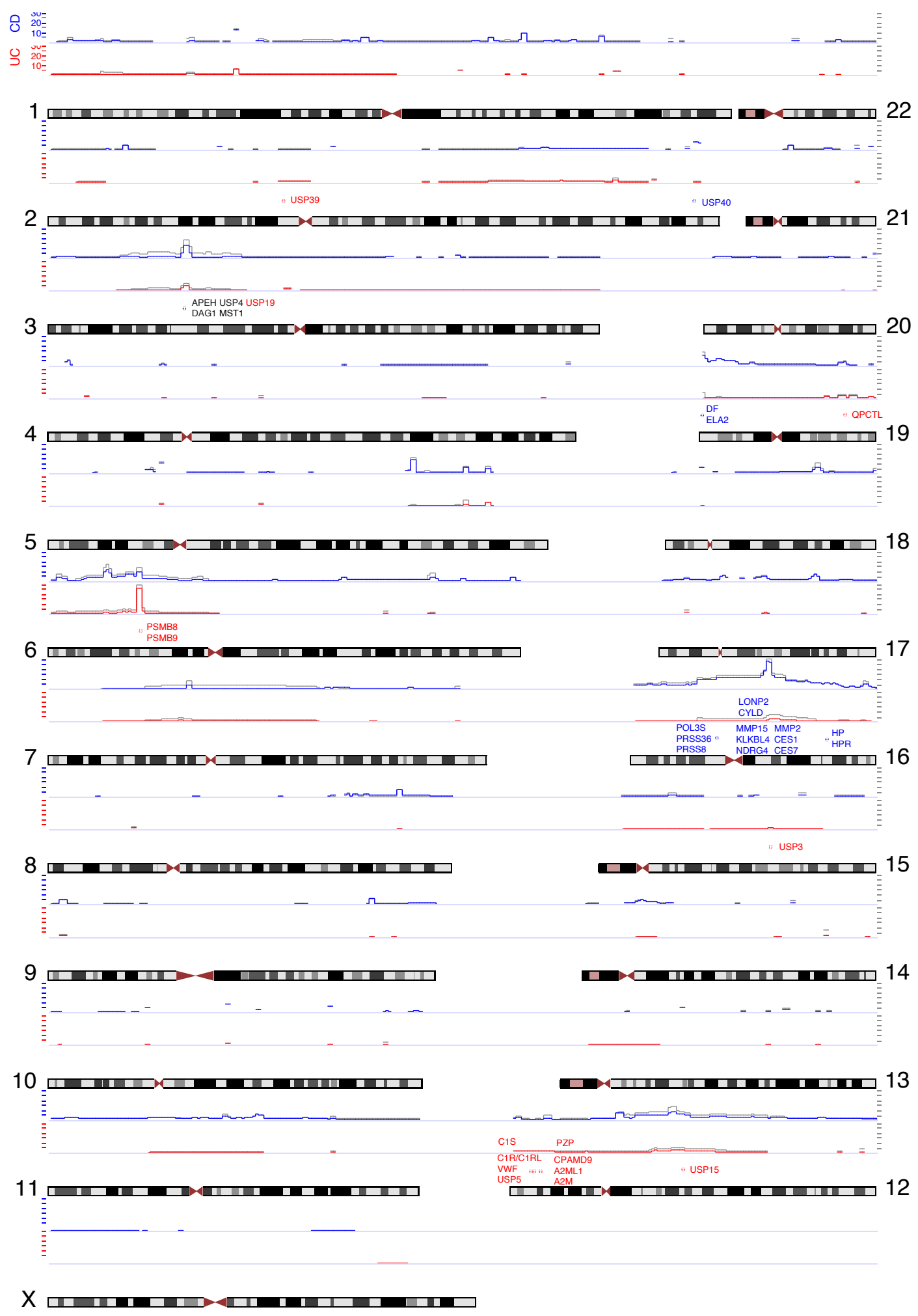

Supplement: Figure S1 — Chromosome plot of the number of studies covering different genomic regions and corresponding numbers of positive studies, presented separately for CD and UC. The total number of performed studies is shown in grey, separately for CD (upper track) and UC (lower track), the number of positive studies reporting a genetic association with CD in blue (upper track) and the number of positive studies reporting a genetic association with UC in red (lower track). Top ranked 20 CD and UC P/PI genes are specified in the figure in blue if associated with CD, in red if associated with UC, in black if associated with both phenotypes. Critical regions defined as before were processed in 1 Mb bins with a perl script and the data was visualized using UCSC Genome Graphs (http://genome.ucsc.edu/cgi-bin/hgGenome). (PDF) [file pone.0024106.s001.pdf]

A

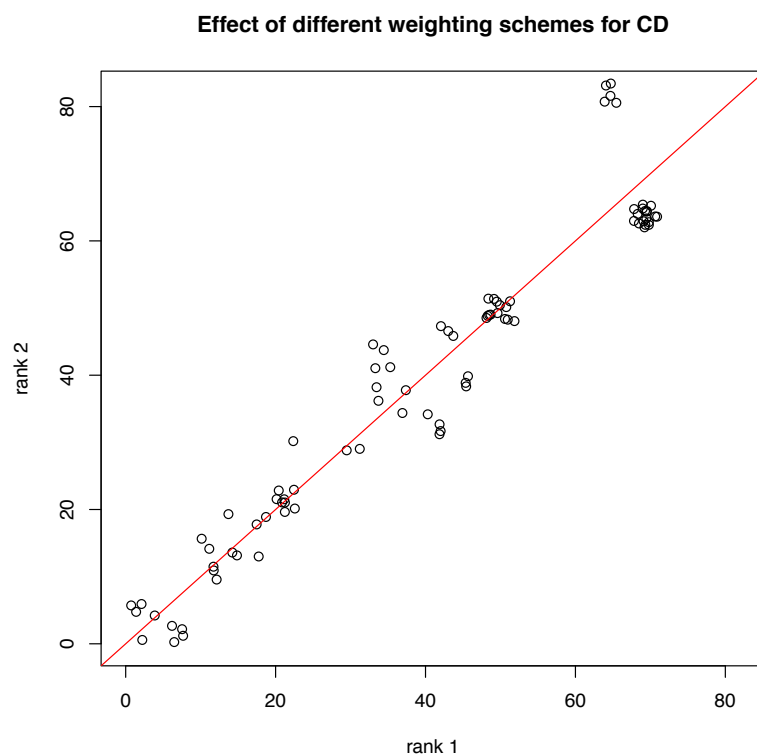

B

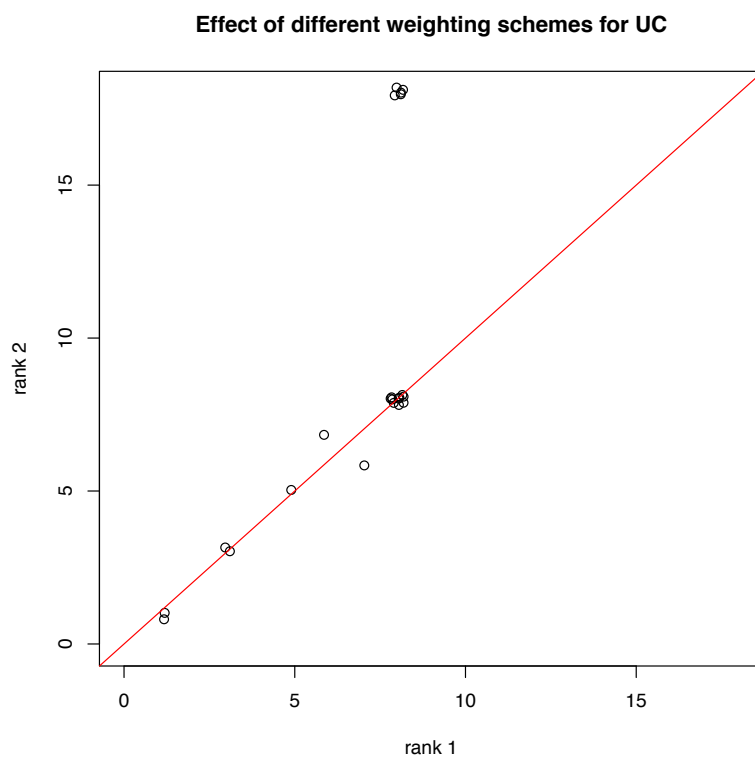

Supplement: Figure S3 — Ranking of P/PI genes in CD and UC with different weighting factors of types of genetic studies. Ranks obtained for CD (panel A) and UC (panel B) applying the original weighting factors set at Cstudy type = 1.00 for GWAS, replication of GWAS and candidate gene studies, Cstudy type = 0.5 for candidate region studies, and Cstudy type = 0.33 for genome-wide linkage scans (rank 1, x-axis) plotted against ranks obtained with an alternate scheme using weighting factors set at Cstudy type = 1.00 for GWAS, replication of GWAS and candidate gene studies, Cstudy type = 0.75 for candidate region studies and Cstudy type = 0.33 for genome-wide linkage scans (rank 2, y-axis). (PDF) [file pone.0024106.s003.pdf]
