# Supplementary material for: Genetic Evidence Supporting the Association of Protease and Protease Inhibitor Genes with Inflammatory Bowel Disease: A Systematic Review
Source: PLoS One. 2011 Sep 8;6(9):e24106. doi: 10.1371/journal.pone.0024106 (PMC3169567; doi:10.1371/journal.pone.0024106)
Supplement: Figure S2 — “GeneRank” Sensitivity assay. Original ranks of P/PI genes on the x-axis are plotted against ranks yielded after omission of GWAS in sensitivity analyses on the y-axis for CD (Panel A) and UC (Panel B). (PDF) [file pone.0024106.s002.pdf]

A

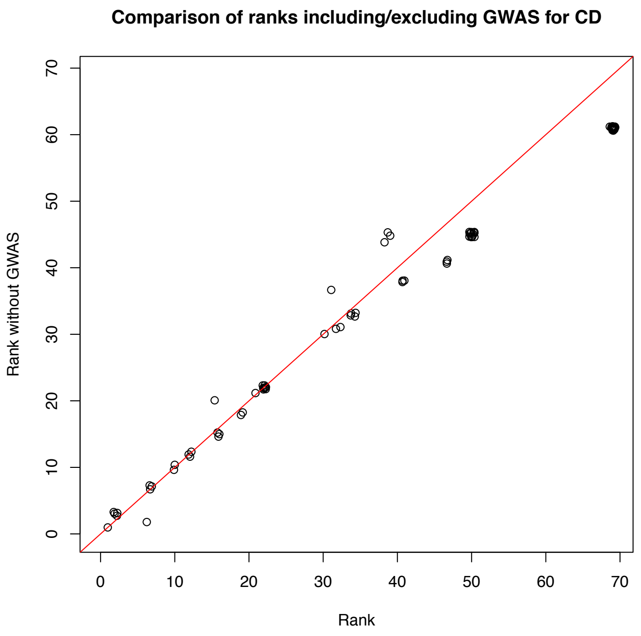

Comparison of ranks including/excluding GWAS for CD
Rank without GWAS
0
10
20
30
40
50
60
70
Rank

B

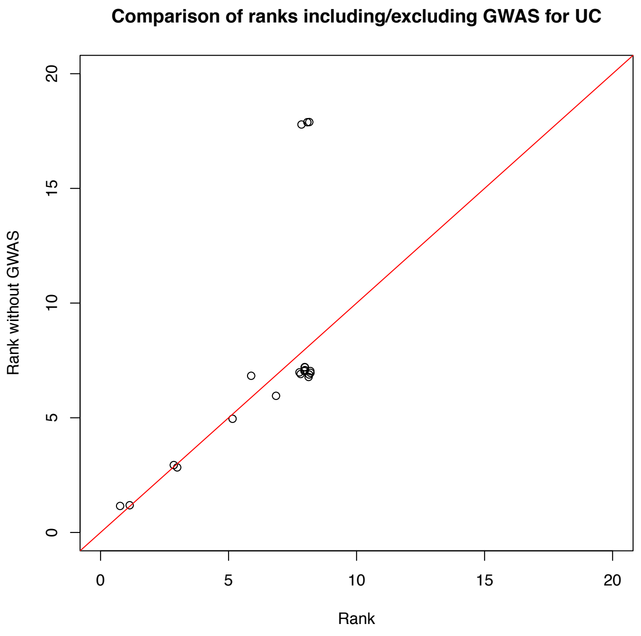

Comparison of ranks including/excluding GWAS for UC
Rank without GWAS
0
5
10
15
20
Rank
