# Supplementary material for: Genetic Evidence Supporting the Association of Protease and Protease Inhibitor Genes with Inflammatory Bowel Disease: A Systematic Review
Source: PLoS One. 2011 Sep 8;6(9):e24106. doi: 10.1371/journal.pone.0024106 (PMC3169567; doi:10.1371/journal.pone.0024106)
Supplement: Table S3 — All proteases and protease inhibitors fulfilling the pre-defined thresholds for Crohn's disease (evidence score >50 and at least 2 positive studies). (DOC) [file pone.0024106.s006.doc]

**Supporting Table 3: All proteases and protease inhibitors fulfilling the pre-defined thresholds for Crohn’s disease.**

| **Rank** | **Protease / protease inhibitor** | **Gene symbol** | **Genomic location^1^** | **Genome-wide association scans (%)^2^** | **Replication of genome-wide association scans (%)^2^** | **Candidate gene studies (%)^2^** | **Candidate region studies (%)^2^** | **Genome-wide linkage scans (%)^2^** | **Number of positive studies (%)** | **Evidence score** |
| --- | --- | --- | --- | --- | --- | --- | --- | --- | --- | --- |
| 1 | CylD protein | CYLD | 16:49333462-49393347 | 5/5 (100%) | 1/1 (100%) |  | 12/14 (86%) | 3/11 (27%) | 21/31 (68%) | 1142 |
| 2 | acylaminoacyl-peptidase | APEH | 3:49686439-49695935 | 1/5 (20%) | 3/3 (100%) |  | 3/6 (50%) | 1/11 (9%) | 8/25 (32%) | 398 |
| 2 | dystroglycan | DAG1 | 3:49482595-49548048 | 1/5 (20%) | 3/3 (100%) |  | 3/6 (50%) | 1/11 (9%) | 8/25 (32%) | 398 |
| 2 | macrophage-stimulating protein | MST1 | 3:49696393-49701110 | 1/5 (20%) | 3/3 (100%) |  | 3/6 (50%) | 1/11 (9%) | 8/25 (32%) | 398 |
| 2 | ubiquitin-specific peptidase 4 | USP4 | 3:49290003-49352519 | 1/5 (20%) | 3/3 (100%) |  | 3/6 (50%) | 1/11 (9%) | 8/25 (32%) | 398 |
| 6 | peroxisomal Lon peptidase | LONP2 | 16:46835712-46944908 | 0/5 (0%) |  |  | 8/9 (89%) | 3/11 (27%) | 11/25 (44%) | 383 |
| 7 | polyserase-3 unit 2 | POL3S | 16:31002246-31007631 | 0/5 (0%) |  |  | 7/8 (88%) | 2/11 (18%) | 9/24 (38%) | 318 |
| 7 | polyserase-2 unit 3 | PRSS36 | 16:31057750-31068888 | 0/5 (0%) |  |  | 7/8 (88%) | 2/11 (18%) | 9/24 (38%) | 318 |
| 7 | prostasin | PRSS8 | 16:31050255-31054320 | 0/5 (0%) |  |  | 7/8 (88%) | 2/11 (18%) | 9/24 (38%) | 318 |
| 10 | complement factor D | DF | 19:810665-814606 | 0/5 (0%) |  | 3/3 (100%) | 0/1 (0%) | 2/11 (18%) | 5/20 (25%) | 312 |
| 10 | elastase-2 | ELA2 | 19:803291-807242 | 0/5 (0%) |  | 3/3 (100%) | 0/1 (0%) | 2/11 (18%) | 5/20 (25%) | 312 |
| 12 | liver carboxylesterase | CES1 | 16:54394267-54424576 | 0/5 (0%) |  |  | 6/7 (86%) | 3/11 (27%) | 9/23 (39%) | 284 |
| 12 | carboxylesterase 7 | CES7 | 16:54437629-54466783 | 0/5 (0%) |  |  | 6/7 (86%) | 3/11 (27%) | 9/23 (39%) | 284 |
| 12 | matrix metallopeptidase-2 | MMP2 | 16:54070589-54098101 | 0/5 (0%) |  |  | 6/7 (86%) | 3/11 (27%) | 9/23 (39%) | 284 |
| 15 | ubiquitin-specific peptidase 40 | USP40 | 2:234048905-234134623 | 2/5 (40%) | 2/2 (100%) |  |  | 0/11 (0%) | 4/18 (22%) | 280 |
| 16 | plasma kallikrein-like protein 4 | KLKBL4 | 16:56871403-56886444 | 0/5 (0%) |  |  | 5/6 (83%) | 2/11 (18%) | 7/22 (32%) | 220 |
| 16 | MT MMP | MMP15 | 16:56616783-56638303 | 0/5 (0%) |  |  | 5/6 (83%) | 2/11 (18%) | 7/22 (32%) | 220 |
| 16 | NDRG4 protein | NDRG4 | 16:57055118-57105024 | 0/5 (0%) |  |  | 5/6 (83%) | 2/11 (18%) | 7/22 (32%) | 220 |
| 19 | haptoglobin-1 | HP | 16:70646009-70652458 | 0/5 (0%) |  | 1/1 (100%) | 2/2 (100%) | 2/11 (18%) | 5/19 (26%) | 212 |
| 19 | haptoglobin-related protein | HPR | 16:70654624-70668645 | 0/5 (0%) |  | 1/1 (100%) | 2/2 (100%) | 2/11 (18%) | 5/19 (26%) | 212 |
| 21 | complement component C3 | C3 | 19:6628846-6671662 | 1/5 (20%) |  | 1/1 (100%) | 1/2 (50%) | 2/11 (18%) | 5/19 (26%) | 157 |
| 22 | carboxylesterase 2 | CES2 | 16:65525848-65536473 | 0/5 (0%) |  |  | 2/2 (100%) | 3/11 (27%) | 5/18 (28%) | 127 |
| 22 | carboxylesterase 3 | CES3 | 16:65552639-65566553 | 0/5 (0%) |  |  | 2/2 (100%) | 3/11 (27%) | 5/18 (28%) | 127 |
| 22 | chymopasin | CTRL | 16:66520975-66523266 | 0/5 (0%) |  |  | 2/2 (100%) | 3/11 (27%) | 5/18 (28%) | 127 |
| 22 | membrane-bound dipeptidase-2 | DPEP2 | 16:66578798-66590857 | 0/5 (0%) |  |  | 2/2 (100%) | 3/11 (27%) | 5/18 (28%) | 127 |
| 22 | membrane-bound dipeptidase-3 | DPEP3 | 16:66567068-66571953 | 0/5 (0%) |  |  | 2/2 (100%) | 3/11 (27%) | 5/18 (28%) | 127 |
| 22 | hypothetical protein flj40219 | MER033212 | 16:65552639-65566552 | 0/5 (0%) |  |  | 2/2 (100%) | 3/11 (27%) | 5/18 (28%) | 127 |
| 22 | hypothetical protein flj37464 | MER033240 | 16:65580134-65601162 | 0/5 (0%) |  |  | 2/2 (100%) | 3/11 (27%) | 5/18 (28%) | 127 |
| 22 | proteasome catalytic subunit 2i | PSMB10 | 16:66525913-66528254 | 0/5 (0%) |  |  | 2/2 (100%) | 3/11 (27%) | 5/18 (28%) | 127 |
| 30 | ubiquitin-specific peptidase 15 | USP15 | 12:60940454-61086165 | 0/5 (0%) |  |  | 4/7 (57%) | 1/11 (9%) | 5/23 (22%) | 117 |
| 30 | elastase-1 | ELA1 | 12:15675183-50026730 | 1/5 (20%) |  |  | 3/6 (50%) | 2/11 (18%) | 6/22 (27%) | 107 |
| 32 | dihydro-orotase (N-terminal unit) | CAD | 2:27293762-27320158 | 0/5 (0%) | 1/1 (100%) |  |  | 1/11 (9%) | 2/17 (12%) | 103 |
| 32 | ubiquitin-specific peptidase 19 | USP19 | 3:49120468-49133316 | 0/5 (0%) | 1/1 (100%) |  | 0/3 (0%) | 1/11 (9%) | 2/20 (10%) | 103 |
| 34 | Atp23 peptidase | XRCC6BP1 | 12:56621627-56637318 | 1/5 (20%) |  |  | 3/7 (43%) | 0/11 (0%) | 4/23 (17%) | 84 |
| 35 | chymotrypsin B | CTRB1 | 16:73810372-73816316 | 1/5 (20%) |  |  | 1/1 (100%) | 2/11 (18%) | 4/17 (24%) | 82 |
| 35 | RECK protein inhibitor unit 1 (SLCO2B1 protein) | SLCO2B1 | 11:74539809-74594945 | 1/5 (20%) |  |  | 1/1 (100%) | 2/11 (18%) | 4/17 (24%) | 82 |
| 37 | Desert hedgehog protein | DHH | 12:47769475-47774869 | 0/5 (0%) |  |  | 3/6 (50%) | 1/11 (9%) | 4/22 (18%) | 78 |
| 37 | SENP1 peptidase | SENP1 | 12:46722948-46785886 | 0/5 (0%) |  |  | 3/6 (50%) | 1/11 (9%) | 4/22 (18%) | 78 |
| 37 | LOC144757 peptidase (Homo sapiens) | TMPRSS12 | 12:49523015-49567928 | 0/5 (0%) |  |  | 3/6 (50%) | 1/11 (9%) | 4/22 (18%) | 78 |
| 40 | hepsin | HPN | 19:40223250-40249315 | 1/5 (20%) |  |  | 1/1 (100%) | 0/11 (0%) | 2/17 (12%) | 70 |
| 40 | Xaa-Pro dipeptidase (eukaryote) | PEPD | 19:38569699-38704522 | 1/5 (20%) |  |  | 1/1 (100%) | 0/11 (0%) | 2/17 (12%) | 70 |
| 40 | Mername-AA103 peptidase | QPCTL | 19:50887772-50898426 | 1/5 (20%) | 0/2 (0%) |  | 1/1 (100%) | 0/11 (0%) | 2/19 (11%) | 70 |
| 43 | matrix metallopeptidase-19 | MMP19 | 12:54515511-54523002 | 0/5 (0%) |  |  | 3/7 (43%) | 1/11 (9%) | 4/23 (17%) | 67 |
| 43 | proliferation-association protein 1 | PA2G4 | 12:54784628-54793912 | 0/5 (0%) |  |  | 3/7 (43%) | 1/11 (9%) | 4/23 (17%) | 67 |
| 43 | ubiquitin-specific peptidase 52 | USP52 | 12:54996988-55014017 | 0/5 (0%) |  |  | 3/7 (43%) | 1/11 (9%) | 4/23 (17%) | 67 |
| 46 | abhydrolase domain-containing protein 4 | ABHD4 | 14:22136986-22151097 | 0/5 (0%) |  |  | 1/1 (100%) | 2/11 (18%) | 3/17 (18%) | 62 |
| 46 | calpain-5 | CAPN5 | 11:76455640-76514844 | 0/5 (0%) |  |  | 1/1 (100%) | 2/11 (18%) | 3/17 (18%) | 62 |
| 46 | chymase (human-type) | CMA1 | 14:24044552-24047311 | 0/5 (0%) |  |  | 1/1 (100%) | 2/11 (18%) | 3/17 (18%) | 62 |
| 46 | cathepsin G | CTSG | 14:24112564-24115306 | 0/5 (0%) |  |  | 1/1 (100%) | 2/11 (18%) | 3/17 (18%) | 62 |
| 46 | granzyme B, human-type | GZMB | 14:24170017-24173313 | 0/5 (0%) |  |  | 1/1 (100%) | 2/11 (18%) | 3/17 (18%) | 62 |
| 46 | granzyme H | GZMH | 14:24145534-24148704 | 0/5 (0%) |  |  | 1/1 (100%) | 2/11 (18%) | 3/17 (18%) | 62 |
| 46 | protein phosphatase methylesterase 1 | MER037853 | 11:73559946-73644654 | 0/5 (0%) |  |  | 1/1 (100%) | 2/11 (18%) | 3/17 (18%) | 62 |
| 46 | membrane-type matrix metallopeptidase-1 | MMP14 | 14:22375633-22386642 | 0/5 (0%) |  |  | 1/1 (100%) | 2/11 (18%) | 3/17 (18%) | 62 |
| 46 | Kae1 putative peptidase | OSGEP | 14:19985056-19993038 | 0/5 (0%) |  |  | 1/1 (100%) | 2/11 (18%) | 3/17 (18%) | 62 |
| 46 | proteasome catalytic subunit 3 | PSMB5 | 14:22564907-22573959 | 0/5 (0%) |  |  | 1/1 (100%) | 2/11 (18%) | 3/17 (18%) | 62 |
| 46 | 26S proteasome non-ATPase regulatory subunit 7 | PSMD7 | 16:72888182-72897685 | 0/5 (0%) |  |  | 1/1 (100%) | 2/11 (18%) | 3/17 (18%) | 62 |
| 46 | colligin 1 | SERPINH1 | 11:74950818-74961492 | 0/5 (0%) |  |  | 1/1 (100%) | 2/11 (18%) | 3/17 (18%) | 62 |
| 46 | chromatin-specific transcription elongation factor 140 kDa subunit | SUPT16H | 14:20889478-20922265 | 0/5 (0%) |  |  | 1/1 (100%) | 2/11 (18%) | 3/17 (18%) | 62 |
| 46 | ubiquitin-specific peptidase 31 | USP31 | 16:22986804-23068092 | 0/5 (0%) |  |  | 2/4 (50%) | 2/11 (18%) | 4/20 (20%) | 62 |
| 60 | peptidase Clp (type 3) | CLPP | 19:6312463-6319915 | 1/5 (20%) |  |  | 1/2 (50%) | 2/11 (18%) | 4/18 (22%) | 57 |
| 60 | dipeptidyl-peptidase 9 | DPP9 | 19:4626238-4674875 | 1/5 (20%) |  |  | 1/2 (50%) | 2/11 (18%) | 4/18 (22%) | 57 |
| 60 | EGF-like module containing mucin-like hormone receptor-like 1 | EMR1 | 19:6838582-6903102 | 1/5 (20%) |  |  | 1/2 (50%) | 2/11 (18%) | 4/18 (22%) | 57 |
| 60 | EGF-like module containing mucin-like hormone receptor-like 4 | EMR4 | 19:6908004-6941857 | 1/5 (20%) |  |  | 1/2 (50%) | 2/11 (18%) | 4/18 (22%) | 57 |
| 60 | PIM1 peptidase | PRSS15 | 19:5642845-5671176 | 1/5 (20%) |  |  | 1/2 (50%) | 2/11 (18%) | 4/18 (22%) | 57 |
| 65 | A430081C19RIK (Mus musculus)-type protein | AGBL2 | 11:47637721-47692878 | 0/5 (0%) |  |  | 1/1 (100%) | 1/11 (9%) | 2/17 (12%) | 53 |
| 65 | isoaspartyl dipeptidase (threonine type) | ASRGL1 | 11:61861501-61917454 | 0/5 (0%) |  |  | 1/1 (100%) | 1/11 (9%) | 2/17 (12%) | 53 |
| 65 | calpain-1 | CAPN1 | 11:64705919-64736052 | 0/5 (0%) |  |  | 1/1 (100%) | 1/11 (9%) | 2/17 (12%) | 53 |
| 65 | cystatin E/M | CST6 | 11:65536038-65537551 | 0/5 (0%) |  |  | 1/1 (100%) | 1/11 (9%) | 2/17 (12%) | 53 |
| 65 | family I29 unassigned peptidase inhibitors | CTSF | 11:66087512-66092623 | 0/5 (0%) |  |  | 1/1 (100%) | 1/11 (9%) | 2/17 (12%) | 53 |
| 65 | cathepsin W | CTSW | 11:65403860-65407788 | 0/5 (0%) |  |  | 1/1 (100%) | 1/11 (9%) | 2/17 (12%) | 53 |
| 65 | dipeptidyl-peptidase III | DPP3 | 11:66004456-66057660 | 0/5 (0%) |  |  | 1/1 (100%) | 1/11 (9%) | 2/17 (12%) | 53 |
| 65 | thrombin (F2 protein) | F2 | 11:46697331-46717631 | 0/5 (0%) |  | 0/1 (0%) | 1/1 (100%) | 1/11 (9%) | 2/18 (11%) | 53 |
| 65 | NAALADASE L peptidase | NAALADL1 | 11:64568873-64582585 | 0/5 (0%) |  |  | 1/1 (100%) | 1/11 (9%) | 2/17 (12%) | 53 |
| 65 | otubain-1 | OTUB1 | 11:63509901-63522468 | 0/5 (0%) |  |  | 1/1 (100%) | 1/11 (9%) | 2/17 (12%) | 53 |
| 65 | pepsin A | PGA3 | 11:60746396-60755755 | 0/5 (0%) |  |  | 1/1 (100%) | 1/11 (9%) | 2/17 (12%) | 53 |
| 65 | pepsin A4 | PGA4 | 11:60746397-60755755 | 0/5 (0%) |  |  | 1/1 (100%) | 1/11 (9%) | 2/17 (12%) | 53 |
| 65 | pepsin A5 | PGA5 | 11:60765245-60775492 | 0/5 (0%) |  |  | 1/1 (100%) | 1/11 (9%) | 2/17 (12%) | 53 |
| 65 | pro-eosinophil major basic protein | PRG2 | 11:56910843-56914688 | 0/5 (0%) |  |  | 1/1 (100%) | 1/11 (9%) | 2/17 (12%) | 53 |
| 65 | family I63 unassigned peptidase inhibitor homologues | PRG3 | 11:56900819-56905199 | 0/5 (0%) |  |  | 1/1 (100%) | 1/11 (9%) | 2/17 (12%) | 53 |
| 65 | prenyl peptidase 1 (protein sequence corrected by use of MEROPS EST alignment) | RCE1 | 11:66367459-66370593 | 0/5 (0%) |  |  | 1/1 (100%) | 1/11 (9%) | 2/17 (12%) | 53 |
| 65 | C1 inhibitor | SERPING1 | 11:57121603-57138902 | 0/5 (0%) |  |  | 1/1 (100%) | 1/11 (9%) | 2/17 (12%) | 53 |
| 65 | ubiquitin-specific peptidase 32 | USP32 | 17:55609473-55824368 | 0/5 (0%) |  |  | 1/1 (100%) | 1/11 (9%) | 2/17 (12%) | 53 |

^1^ Chromosome: start and end boundaries (base pairs; NCBI Build 36 coordinates)

^2^ Number of positive studies / total studies, percentage of positive studies of respective study type in brackets; empty cells indicate no evidence available for the specific gene and the respective study type
